# Emergomycosis, an Emerging Systemic Mycosis in Immunocompromised Patients: Current Trends and Future Prospects

**DOI:** 10.3389/fmed.2021.670731

**Published:** 2021-04-23

**Authors:** Arghadip Samaddar, Anuradha Sharma

**Affiliations:** Department of Microbiology, All India Institute of Medical Sciences, Jodhpur, India

**Keywords:** emergomycosis, emergomyces, dimorphic fungi, endemic mycoses, AIDS-related mycosis, antifungal drug

## Abstract

Recently, the global emergence of emergomycosis, a systemic fungal infection caused by a novel dimorphic fungus *Emergomyces* species has been observed among immunocompromised individuals. Though initially classified under the genus *Emmonsia*, a taxonomic revision in 2017 based on DNA sequence analyses placed five *Emmonsia*-like fungi under a separate genus *Emergomyces*. These include *Emergomyces pasteurianus, Emergomyces africanus, Emergomyces canadensis, Emergomyces orientalis*, and *Emergomyces europaeus*. *Emmonsia parva* was renamed as *Blastomyces parvus*, while *Emmonsia crescens* and *Emmonsia sola* remained within the genus *Emmonsia* until a taxonomic revision in 2020 placed both the species under the genus *Emergomyces*. However, unlike other members of the genus, *Emergomyces crescens* and *Emergomyces sola* do not cause disseminated disease. The former causes adiaspiromycosis, a granulomatous pulmonary disease, while the latter has not been associated with human disease. So far, emergomycosis has been mapped across four continents: Asia, Europe, Africa and North America. However, considering the increasing prevalence of HIV/AIDS, it is presumed that the disease must have a worldwide distribution with many cases going undetected. Diagnosis of emergomycosis remains challenging. It should be considered in the differential diagnosis of histoplasmosis as there is considerable clinical and histopathological overlap between the two entities. Sequencing the internal transcribed spacer region of ribosomal DNA is considered as the gold standard for identification, but its application is compromised in resource limited settings. Serological tests are non-specific and demonstrate cross-reactivity with *Histoplasma* galactomannan antigen. Therefore, an affordable, accessible, and reliable diagnostic test is the need of the hour to enable its diagnosis in endemic regions and also for epidemiological surveillance. Currently, there are no consensus guidelines for the treatment of emergomycosis. The recommended regimen consists of amphotericin B (deoxycholate or liposomal formulation) for 1–2 weeks, followed by oral itraconazole for at least 12 months. This review elaborates the taxonomic, clinical, diagnostic, and therapeutic aspects of emergomycosis. It also enumerates several novel antifungal drugs which might hold promise in the treatment of this condition and therefore, can be potential areas of future studies.

## Background

Fungal infections are constantly evolving with new genera and species being increasingly implicated in human diseases. Consequently, the diagnosis and management of such infections become challenging. One such infection that has been recently observed among immunocompromised individuals is attributed to a novel dimorphic fungus closely related to *Emmonsia* species (1). Due to taxonomic similarity, they were initially classified under the genus *Emmonsia*, either as *Emmonsia pasteuriana* or *Emmonsia*-like species. However, genetic sequence analysis, later on, found that these fungi belonged to a previously unknown genus and due to their recent global emergence, they were ascribed to a new genus *Emergomyces* and the disease was designated as emergomycosis (2).

## Recent Taxonomy and Classification

Since the 1970's, there has been an emergence of several novel fungi with phylogenetic, and morphological similarities to known members of the *Ajellomycetaceae* family. To resolve the phylogenetic relationships among *Emmonsia* and *Emmonsia*-like fungi, which seem to be closely related to *Blastomyces* spp. and to a lesser extent to other members of *Ajellomycetaceae* family, global collections of *Emmonsia*-like fungi were re-examined using concatenated sequence data of five loci: large subunit rDNA (LSU), internal transcribed spacer (ITS), β-tubulin (TUB2), elongation factor 3 (TEF3), and RNA polymerase II (rPB2) (2–4). This led to a taxonomic revision within the *Ajellomycetaceae* family in 2017, following which five *Emmonsia*-like fungi were placed under a separate genus *Emergomyces*, members of which have been associated with a fatal disseminated mycosis in Asia, Europe, Africa and North America (3). The five distinct species under the genus included *Emergomyces pasteurianus, Emergomyces africanus, Emergomyces canadensis, Emergomyces orientalis*, and *Emergomyces europaeus* (1, 3, 4). *Emmonisa parva* was shifted to the genus *Blastomyces* as *Blastomyces parvus*, while *Emmonsia sola* and *Emmonsia crescens* remained in the genus *Emmonsia*. Initially, *Emergomyces* spp. were differentiated from *Emmonsia* spp. by the presence of small budding yeast cells instead of thick-walled non-replicating adiaspores in their thermotolerant phase (4, 5). However, later it became evident that species within the genus *Emergomyces* can present a spectrum of morphologies from budding yeast cells to adiaspores. Consequently, the genus *Emmonsia* was removed and *Ea. sola* and *Ea. crescens* were re-classified as *Emergomyces* in 2020 after further analyses (6). However, unlike other members of the genus *Emergomyces, Es. crescens* and *Es. sola* do not cause disseminated disease. The former causes adiaspiromycosis, a granulomatous pulmonary disease, while the latter has not been associated with human disease. *Es. pasteurianus* is the most widespread and represents the type species, while *Es. africanus* is one of the most frequent endemic mycosis diagnosed in South Africa. (7) The order *Onygenales* of *Ajellomycetaceae* family currently comprises of seven genera: *Emergomyces, Blastomyces, Histoplasma, Paracoccidioides, Lacazia, Emmonsiellopsis*, and *Helicocarpus* (2, 4, 5, 8–10), as summarized in Table 1.

**Table 1:** Current taxonomic classification and geographic distribution of *Ajellomycetaceae* family (order *Onygenales*) (2, 4, 5, 8–10).

**Genus**	**Species**	**Origin (host)**	**Geographic distribution**
Emergomyces	*Emergomyces pasteurianus*	Skin (human)	Italy, Spain, France, the Netherlands, China, India, Uganda, South Africa
	*Emergomyces africanus*	Skin (human)	South Africa, Zimbabwe, Lesotho
	*Emergomyces canadensis*	Skin, blood (human)	Canada, USA
	*Emergomyces orientalis*	Skin (human)	China
	*Emergomyces europaeus*	Lung (human)	Germany
	*Emergomyces crescens*	Lung (rodent)	UK, USA, Canada
	*Emergomyces sola*	Soil	USA
Blastomyces	*Blastomyces dermatitidis*	Human	USA
	*Blastomyces parvus*	Lung (rodent)	USA
	*Blastomyces helicus*	Lung (feline, dog) Sputum, BAL, CSF, bone marrow, blood (human)	USA, Canada
	*Blastomyces silverae*	Lung, BAL (human)	Canada
	*Blastomyces gilchristi*	Sputum (human)	Canada
	*Blastomyces percursus*	Skin (human)	South Africa, Israel
	*Blastomyces emzantsi*	Human	South Africa
Histoplasma	*Histoplasma capsulatum* var. *capsulatum*	Bone marrow, skin, blood (human)	Worldwide with variable endemicity
	*Histoplasma capsulatum* var. *duboisii*	Skin (human)	Central and West Africa, Madagascar
	*Histoplasma capsulatum* var. *farciminosum*	Horse	Egypt
	*Histoplasma capsulatum sensu stricto*	Human	Colombia
	*Histoplasma mississippiense*	Lung (human)	USA
	*Histoplasma ohiense*	Lung (human)	USA
	*Histoplasma suramericanum*	Lung (human)	Colombia
Paracoccidioides	*Paracoccidioides brasiliensis*	Human	Brazil, Argentina, Venezuela, Mexico
	*Paracoccidioides lutzii*	Human	Brazil
Lacazia	*Lacazia loboi*	Skin and subcutaneous tissues (Human, bottle-nosed dolphins)	Central and South America, USA, Canada, France, Netherlands, Germany, Greece, South Africa
Emmonsiellopsis	*Emmonsiellopsis terrestris*	Soil	USA, Spain
	*Emmonsiellopsis coralliformis*	Soil	Spain
Helicocarpus	*Helicocarpus griseus*	Gazelle dung, soil	Algeria

*BAL, Bronchoalveolar lavage; CSF, Cerebrospinal fluid*.

## Geographical Distribution

To date, emergomycosis has been reported from four continents: Asia, Africa, Europe and North America (1, 2, 4). However, several authors suggest that the disease might have a global prevalence. The genus *Emergomyces* consists of five species: *Es. pasteurianus*, the type species, has been reported from Asia (China (11, 12) and India (13, 14)), Europe (Italy (15), France (16), Spain (17), and the Netherlands (18)), and Africa (Uganda (19) and South Africa (2)), *Es. africanus* from South Africa, (2) Zimbabwe, and Lesotho (20), *Es. canadensis* from Canada (Saskatchewan) and the United States (Colorado and New Mexico) (21), *Es. orientalis* from China (Shanxi), (22) and *Es. europaeus* from Germany (23). The most extensive burden of the disease has been observed among HIV-infected patients of South Africa where it has been reported from six of the nine provinces and most cases are attributed to *Es. africanus* (24). All South African cases of emergomycosis were diagnosed after the introduction of broad-range fungal PCR in 2013. Thus, the apparent clustering of cases and the emergence of *Es. africanus* in South Africa may simply represent accurate identification of the causative agent rather than introduction of a novel dimorphic fungal pathogen. There is a possibility that many cases of emergomycosis might have gone missed due to lack of awareness regarding the disease and misidentification of the causative agent. The latter hypothesis is supported by the dramatic increase in the number of cases of disseminated emergomycosis with a relative decline in the number of histoplasmosis cases in South Africa, following the introduction of molecular identification techniques (25, 26). All the South African cases were adults with advanced HIV disease (median CD4+ T cell count 16 cells/μL) and had extensive cutaneous involvement (27). Although country-level surveillance data is lacking, a clinical and laboratory surveillance study at public hospitals in Cape Town, South Africa, documented 17 culture proven cases of emergomycosis over a period of 15 months (28). Detection of *Es. africanus* DNA in 30% of soils samples from the Western Cape Province (7) and in 10% of air samples from Cape Town (26), indicate that the fungus might have an ecological niche. However, to date, it has not been successfully isolated in culture from the environment. Also, natural infections in animals have not yet been documented (29).

## Virulence and Pathogenesis

Infection with *Emergomyces* spp. is presumed to occur through inhalation of conidia present in soil, followed by *in vivo* transformation to a yeast-like phase that is capable of extrapulmonary dissemination in susceptible hosts. Little is known regarding the virulence factors of this group of dimorphic fungi and the pathogenesis of emergomycosis. The virulence factor genes present in other members of the *Ajellomycetaceae* family are conserved in *Es. pasteurianus* and *Es. africanus* (8). The evolutionary transitions that this family of fungi has undergone, allow for their adaptation, infection and virulence in humans, and interactions with other eukaryotes in the environment that may help in maintaining their pathogenic potential in mammalian hosts. Besides, selection pressures in the environment are responsible for the emergence and maintenance of traits that confer upon them the capacity to survive in animal hosts. Munoz et al. (8) found significant expansions of the number of the fungal-specific protein kinase (FunK1) family, transcription factors and other genes associated with the regulation of gene expression in *Ajellomycetaceae*, highlighting their possible role in virulence and invasive disease. *Es. pasteurianus, Es. africanus*, and *Es. europaeus* (but not *Es. canadensis* and *Es. orientalis*) produce urease enzyme, a known virulence factor that impairs fungal clearance and leads to increased fungal burden at the primary site of infection (4, 30, 31). Lerm et al. (30) reported that the urease produced by *Es. africanus* is functionally similar to that of *Cryptococcus neoformans*, indicating its possible role in persistence of primary infection and subsequent extrapulmonary dissemination in susceptible hosts. Schwartz et al. (7) demonstrated that intraperitoneal inoculation of BALB/c and C57BL/6 mice with *Es. africanus* in doses of 10^6^ conidia resulted in significantly higher mortality in C57BL/6 mice as compared with BALB/c mice. This observation suggests that genetic background possibly has an influence on host susceptibility to the organism.

## Clinical Presentation

**Figure 1:**
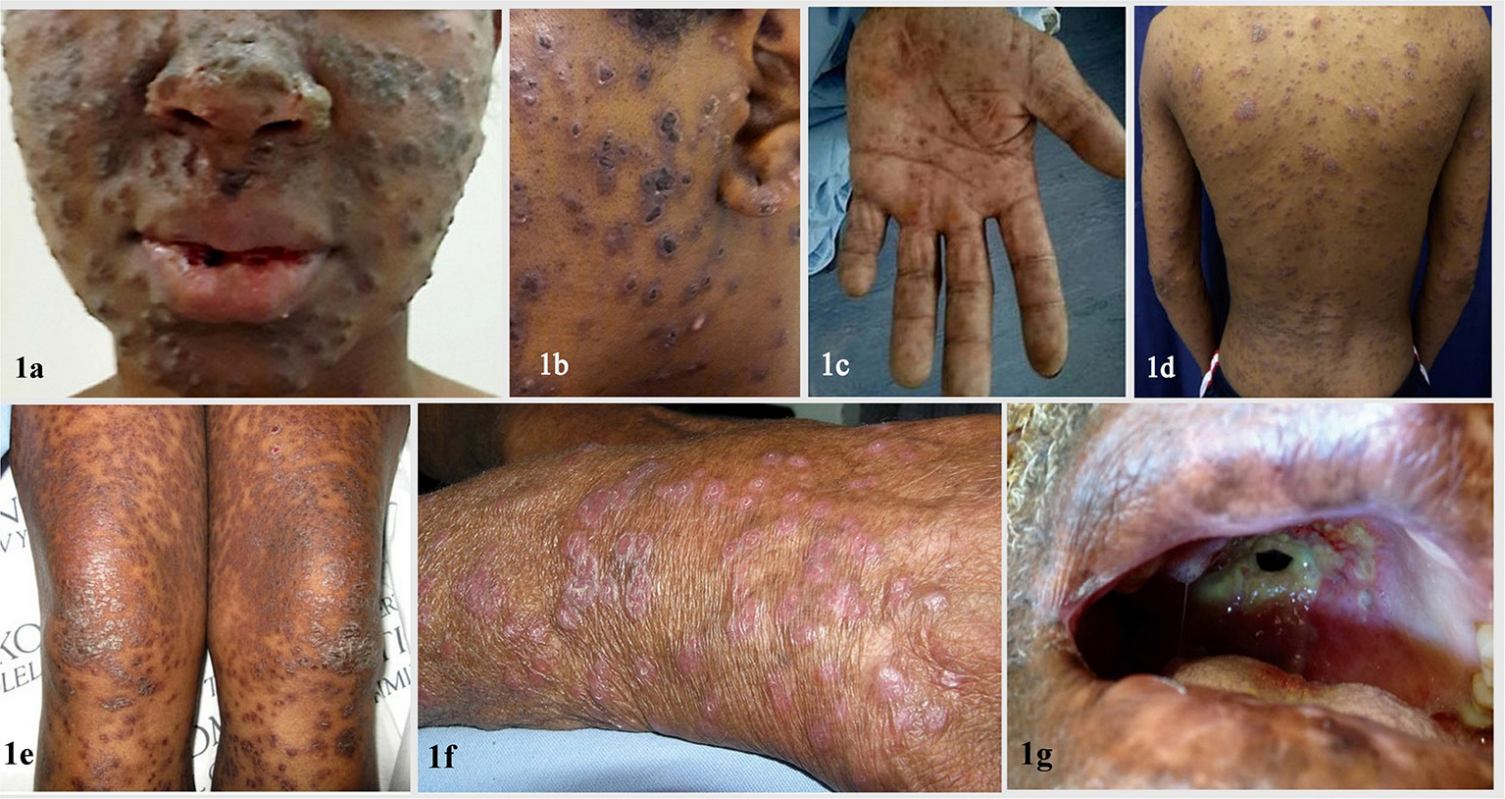
Clinical images depicting various mucocutaneous manifestations of disseminated emergomycosis including ulcerated and crusted facial plaques and nodules **(a,b)**, erythematous scaly lesions **(c–f)**, palmar involvement **(c)**, and oroantral fistula **(g)** Reproduced from references (27) (with permission) & (32) (published under Creative Commons Attribution-Non-Commercial-NoDerivatives 4.0 International License).

The primary route of infection is presumed to be inhalation of airborne conidia released from saprophytic mycelia in soil (7). Inside the human host, these conidia are transformed into yeast-like cells capable of replication and extrapulmonary dissemination. All reported cases of disseminated infection caused by *Emergomyces* spp. have occurred in immunocompromised adults, the vast majority of which had advanced HIV infection. Other underlying risk factors include neutropenia, solid organ transplantation, hematological malignancies, and use of immunosuppressive drugs (3). Earlier reports of the disease in immunocompetent patients from South Africa were, in fact, due to another novel dimorphic fungus endemic to the region, *Blastomyces percursus* (2). In a single case of the disease caused by *Es. orientalis*, there was no underlying immunodeficiency apart from type 2 diabetes mellitus (22). Emergomycosis is a multisystem disease with involvement of skin, lungs, liver, spleen, bone marrow, lymph nodes, brain, and cervix. Schwartz et al. (27) in a study from South Africa reported that 96% of patients with disseminated disease had cutaneous lesions; all of them had very low CD4+ T cell counts (median CD4 count 16 cells/mm^3^) and were profoundly anemic. Cutaneous involvement occurs in the form of umbilicated papules, nodules, ulcers, verrucuous lesions, crusted plaques, and erythema (27, 32). Skin lesions with varying morphologies can be observed in individual patients (Figure 1).

Pulmonary involvement occurs in the form of diffuse and focal reticulonodular infiltrates, consolidations, lobar atelectasis, effusions, and hilar lymphadenopathy. Limited pulmonary disease has been observed in the sole reported case of the disease caused by *Es. europaeus* (23). The various clinical manifestations of disseminated emergomycosis are summarized in Table 2.

**Table 2:** Clinical manifestations of emergomycosis (3, 21, 27, 32).

**System involved**	**Clinical manifestations, laboratory, and imaging findings**
Skin	Umbilicated papules, nodules, ulcers, verrucuous lesions, crusted hyperkeratotic plaques, erythema
Respiratory system	Upper respiratory: epistaxis, nasal congestion, oroantral fistula
	Lower respiratory: pneumonia, lobar atelectasis!!break Imaging findings: diffuse and focal reticulonodular infiltrates, consolidations, lobar atelectasis, effusions, and hilar lymphadenopathy
Hematologic system	Anemia, thrombocytopenia
Central nervous system	Altered mental status, headache, seizure, ataxia, loss of visual acuity, personality changes Laboratory findings: CSF pleocytosis, low CSF glucose, elevated CSF protein
Gastrointestinal system	Laboratory findings: elevated levels of serum bilirubin, alkaline phosphatise, alanine aminotransferase, aspartate aminotransferase, and gamma-glutamyl transferase!!break Imaging findings: hepatomegaly, abnormal echogenicity of liver, splenic lesions, lymphadenopathy, abdominal mass
Genital system	Endocervical mass

*CSF, Cerebrospinal fluid*.

## Differential Diagnosis

*Emergomyces* is a mimicking fungus and many initial cases were misdiagnosed as histoplasmosis, cryptococcosis, sporotrichosis, and blastomycosis (24). The cutaneous lesions of emergomycosis should be differentiated from varicella, scrofuloderma, papular eruption of HIV, cutaneous lesions of secondary syphilis, drug reactions, Kaposi sarcoma, guttate psoriasis, and pyoderma gangrenosum. Pulmonary lesions on chest radiograph may mimic pulmonary tuberculosis (27). Moreover, the morphological features of this fungus can be sometimes misleading. The yeast phase of *Es. africanus* closely resembles *Histoplasma capsulatum*, while that of *Es. orientalis* resembles *Blastomyces dermatitidis* (4). Histopathology can detect yeasts, but is unable to differentiate between the different fungal genera. Therefore, fungal culture is imperative for differentiating between these dimorphic fungi. However, the mold phase of *Es. africanus* closely resembles *Sporothrix schenckii* on microscopy (32). For all these reasons, molecular techniques like sequencing are considered as the reference tool for identification.

## Diagnosis

Diagnosis of emergomycosis poses a challenge for the clinicians and microbiologists. Studies indicate that three-quarters of patients with emergomycosis get misdiagnosed as tuberculosis and receive treatment for the latter (27). Diagnosis can be made by histopathological examination and fungal culture of affected tissue obtained by biopsy. The recent introduction of molecular techniques has improved the diagnostic accuracy. Blood, skin tissue, bone marrow aspirate and/or trephine biopsy, lymph node aspirate, induced sputum, or bronchoalveolar lavage (BAL) specimens are appropriate for mycological investigations.

### Histopathology

There is considerable overlap between the histopathological features of emergomycosis and histoplasmosis. In fact, these infections are virtually indistinguishable from one another on histopathology, and a definitive diagnosis therefore, requires detailed clinicopathological correlation and the use of fungal culture and molecular identification techniques (33). Hematoxylin-eosin, periodic acid-Schiff, and Gomori's methenamine silver stains can be used to demonstrate the intracellular yeasts with surrounding inflammatory changes in the affected tissue. Histology of skin biopsy specimen shows chronic granulomatous and/or suppurative dermal infiltrates containing histiocytes, multinucleated giant cells, and plasma cells, together with intracytoplasmic narrow-based budding yeasts, measuring 2–5 μm in size (24, 32). Sometimes pseudo-epitheliomatous hyperplasia accompanied by transepidermal and/or transfollicular elimination of the fungal organisms can also be seen. There may be invasion of the dermal nerves. In profoundly immunosuppressed individuals, the host inflammatory response may be minimal to virtually absent, and in such cases, the skin biopsy may appear near-normal (32). Cases occurring as a manifestation of immune reconstitution inflammatory syndrome (IRIS) exhibit a more pronounced mixed dermal inflammatory infiltrate and even microabscess formation (34).

### Fungal Culture

**Figure 2:**
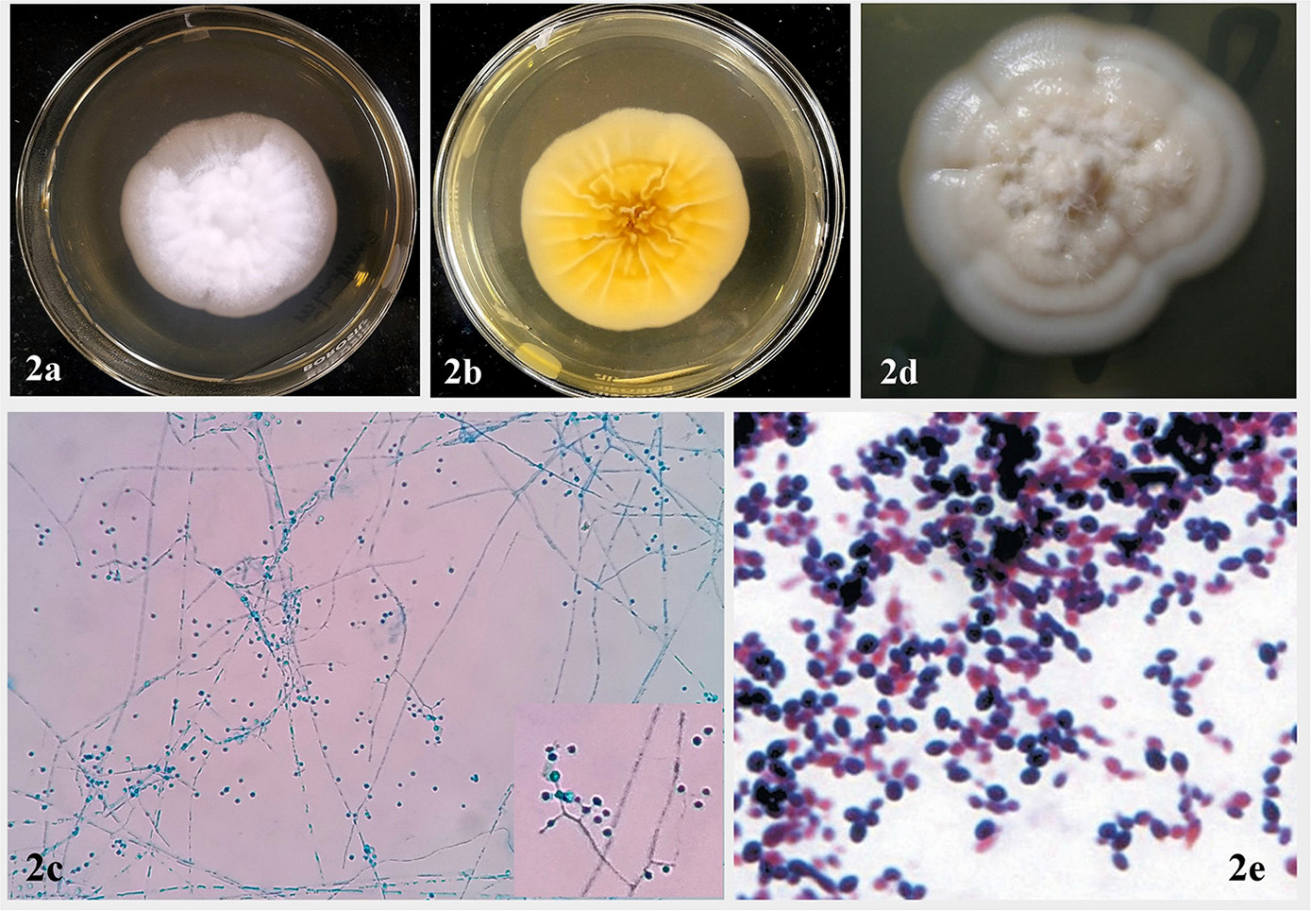
Colony characteristics and microscopic morphology of mycelial phase of *Emergomyces* spp. on SDA after 14 days of incubation at 25°C. **(a)** Obverse showing white to tan, initially glabrous colony, which becomes powdery, slightly raised, and furrowed with age. **(b)** Reverse showing ochraceous-buff to warm buff peripherally. **(c)** Microscopic morphology in lactophenol cotton blue preparation showing “florets” of smooth-walled subglobose conidia borne on slender conidiophores, slightly swollen at the tip and arising at right angles from thin-walled hyaline hyphae (inset) (Courtesy: Advanced Mycology Laboratory, Department of Microbiology, All India Institute of Medical Sciences, Jodhpur, Rajasthan, India). **(d)** Yeast phase of *Emergomyces* species on BHI agar, showing yeast-like, pasty, cerebriform yellowish-white to tan colonies after 2–3 weeks of incubation at 37°C. **(e)** Gram stain morphology of yeast phase showing Gram positive round to oval yeast cells with narrow based budding (Reproduced from references (20) and (22), published under Creative Commons Attribution-Non-Commercial-NoDerivatives 4.0 International License).

*Emergomyces* spp. grow readily on routine mycology media like Sabouraud dextrose agar (SDA), malt extract agar (MEA), or potato dextrose agar (PDA), incubated at 24–30°C. Colonies are yellowish white to tan, initially glabrous, becoming powdery, slightly raised and furrowed, and reach diameters of 2.5–3.5 cm in 2 weeks (Figure 2a). Reverse is ochraceous-buff to warm-buff peripherally (Figure 2b). Microscopic morphology of the mold phase in lactophenol cotton blue preparation exhibits slender conidiophores arising at right angles from thin-walled hyaline hyphae, slightly swollen at the tip, sometimes with short secondary conidiophores bearing “florets” of solitary single-celled subspherical conidia (Figure 2c). For conversion from mold to the yeast phase, mold colony from SDA is subcultured on MEA or brain heart infusion (BHI) agar containing 5% sheep blood and incubated at 35°C. Yellowish-white to tan, pasty, cerebriform colonies appear after 2–3 weeks of incubation (Figure 2d). Gram stained smear prepared from culture reveals small, oval yeast cells with narrow based budding (Figure 2e). The morphological features of mycelial and yeast phases of *Emergomyces* species have been summarized in Table 3.

**Table 3:** Morphological features of yeast and mold phases of *Emergomyces* species (4).

**Species**	**Saprobic phase (24**^****°****^**C)**		**Thermotolerant phase (37**^****°****^**C)**		**Comments**
	**Colony morphology**	**Microscopic morphology**	**Colony morphology**	**Microscopic morphology**	
*Emergomyces pasteurianus*	**Obverse-**Yellowish white, dense, felty to floccose, radially sulcate.!!break **Reverse**-Ochraceous-buff to warm buff peripherally.	Conidiophores with septa at the base and at conidial insertion, cylindrical, or moderately swollen at the tip. Conidia formed singly or in short chains (2–4), subspherical, 0.9–2.8 × 1.8–3.2 μm, smooth to finely roughened. Some chlamydospore-like cells arise terminally on short lateral branches, with thickened walls and often with a median septum	8 mm diameter, yeast-like, cerebriform, yellowish-white.	Hyphae scant, moniliform, some cells becoming giant cells, 5.4–12 μm wide. Yeast cells arise from giant cells or from fragments of swollen conidiophores or hyphae; small yeasts with narrow-based budding, 2.1–5.1 × 1.6–4.2 μm; larger yeasts 5.0–11.2 × 2.4–6.3 μm, with uni- or bipolar budding from narrow or broad bases.	Conversion to yeast is slower (2–3 weeks) and occurs at higher temperature (37°C) than in *Es. africanus* and *Es. europaeus*.
*Emergomyces africanus*	**Obverse**-Yellowish-white, glabrous to floccose centrally, radially sulcate.!!break **Reverse**-Warm-buff to light buff peripherally.	Conidiophores with a septum at the base and at conidial insertion; moderately swollen at the tip with 4–8 conidia borne on narrow pedicels. Conidia single or in short chains (2–4), subspherical, 1.2–3.2 × 1.7–3.8 μm, smooth to finely roughened.	7 mm diameter, yeast-like, cerebriform, yellowish-white.	Hyphae scant. Yeast cells abundant, ovoidal to subspherical, 1.7–5.3 × 0.9–2.2 μm with unipolar budding at a narrow base.	Development of secondary conidiophores leading to a complex cluster of 4–8 conidia and production of small-celled yeasts at 37°C within 1 week.
*Emergomyces canadensis*	**Obverse-** Yellowish white, cottony to glabrous, with tufts of hyphae centrally, radially sulcate.!!break **Reverse**-Ochraceous-buff to warm-buff peripherally.	Conidiophores with septum at the base, cylindrical, or slightly swollen in the middle and at the tip, bearing 1–2 conidia on narrow pedicels. Conidia subspherical, 2.1–3.8 × 1.8–3.4 μm, smooth to slightly roughened.	3 mm diameter, yeast-like, smooth, yellowish-white.	Yeast cells abundant, spherical, 2.2–4.8 μm with uni- or bipolar budding at narrow base. Few short, swollen hyphal elements, and giant cells present.	Closely related to *Es. orientalis*. Urease test is negative, red pigment produced on BHIA, and TSA at 37°C. In *Es. canadensis*, optimal sporulation temperature is 24–27°C, time for transformation to yeast at 37°C is fast (1 week).
*Emergomyces orientalis*	**Obverse-**Yellowish white, felty with hyphal tufts centrally, radially sulcate.!!break **Reverse-**Ochraceous-buff to warm-buff peripherally.	Conidiophores cylindrical or slightly swollen in the middle, with a septum at the base, thin secondary conidiophores present. Conidia subspherical, 1.1–2.8 × 1.7–4.8 μm smooth to slightly roughened.	5 mm diameter, yeast-like, cerebriform, yellowish white.	Hyphal elements scant. Yeast cells spherical, 2.0–4.5 μm diam, with uni- or bipolar budding at a narrow base. Few giant cells present.	Produces conidia at 21°C but not at 24°C. Urease test is negative, red pigment produced on BHIA and TSA at 37°C. Time for transformation to yeast at 37°C is slow (2 weeks).
*Emergomyces europaeus*	**Obverse-** Dense, white, felty to floccose, radially sulcate, glabrous at the margin.!!break **Reverse**-Warm-buff to light buff periphally.	Conidiophores unbranched, with septum at the base, cylindrical to slightly swollen at the tip, bearing one or two subspherical, slightly roughened conidia, measuring 2.9–5.7 × 3.0–5.7 μm	4 mm diameter, yeast-like, pasty, cerebriform, tan	Swollen hyphae and giant cells present; yeast cells ovoidal to subspherical, 2.6–5.9 × 17–3.8 μm with uni- or bipolar budding at a narrow base.	*Es. europaeus* has conidiophores without secondary branches and conidia are larger and more roughened than those of other species. Yeast transformation occurs at a lower temperature (33°C).

*BHIA, Brain heart infusion agar; TSA, Trypticase soy agar*.

### Molecular Identification

Mycologists have traditionally used morphological (phenotypic) characteristics, such as conidiogenesis for fungal identification. It is still being used routinely for classification of fungi at the ordinal or family level in most mycology laboratories. However, morphological features may considerably overlap between different fungal genera and therefore, may not be ideal for speciation. For example, the mycelial form of *Emergomyces* spp. sometimes resembles *Sporothrix schenckii*, another dimorphic fungus causing subcutaneous mycosis in endemic regions (32). Therefore, molecular techniques like polymerase chain reaction (PCR) and sequencing are considered as the gold standard for identification. They can also be employed for direct detection of fungi in clinical and environmental samples. However, the diagnostic accuracy of molecular assays depends on several factors, such as optimal specimen selection, fungal load in specimens, and primer design (pan-fungal or species-specific). It is crucial to differentiate environmental fungal DNA contamination from target fungal DNA in clinical specimens, particularly while using pan-fungal primers (35). At present, there are no commercially available molecular diagnostic tests for *Emergomyces* spp. Recently, Alanio et al. (36) demonstrated excellent sensitivity of a whole nucleic acid-based reverse transcriptase quantitative PCR (RTqPCR) assay for the diagnosis of histoplasmosis and the same platform is being evaluated for emergomycosis.

#### From Culture Isolate

Fungal genomic DNA is extracted from a 20-day-old culture of the isolate using commercially available kits like Zymo ZR fungal/bacterial DNA MiniPrep kit (Zymo Research, Irvine, CA) or MasterPure™ Yeast DNA Purification Kit (Epicentre, Madison, WI, U.S.A.), followed by PCR and sequencing of the *ITS* and *LSU* regions of the ribosomal DNA with primer pairs *ITS5* (5′-GGAAGTAAAAGTCGTAACAAGG-3′) and *ITS4* (5′-TCCTCCGCTTATTGATATGC-3′), and *LR0R* (5′-ACCCGCTGAACTTAAGC-3′) and *LR5* (5′-TCCTGAGGGAAACTTCG-3′), respectively (2, 37). *Candida albicans* ATCC 90028 is included as a quality control (QC) strain during PCR and sequencing of the ITS region.

#### From Tissue Specimen

When fungal cultures are negative or omitted, PCR of fresh, affected tissue specimen with amplification and sequencing of the *ITS* and *LSU* regions can help in establishing the correct diagnosis. The tissue samples are first homogenized in lysis buffer (containing β-mercaptoethanol) with either a homogeniser or by repeatedly passing the finely chopped tissue specimen through a 2-ml syringe with a 22-gauge needle. Samples are then centrifuged and the cell-free supernatant is used as starting material as per the manufacturer's instructions. The extracted DNA concentration is determined spectrophotometrically and a conventional PCR is used to screen samples for the presence of fungal DNA (29). The universal fungal locus *ITS1–5.8S–ITS2* and partial LSU region of rDNA are amplified using primer pairs *ITS5* and *ITS4*, and *LR0R* and *LR5*, respectively, operated under standard PCR conditions as described by Dukik et al. (2) Sequences are determined by capillary electrophoresis and identification to the species level is done with the National Center for Biotechnology Information (NCBI) Basic Local Alignment Search Tool (BLAST) database on the basis of pairwise sequence alignment. It should be noted that *Emergomyces* spp. can cross-react with a commercial *B. dermatitidis* DNA probe (AccuProbe, Hologic, Inc., San Diego, CA, USA) (21).

#### From Formalin-Fixed, Paraffin-Embedded (FFPE) Tissue

Identification of fungi from histopathology sections is limited by failure of species identification. In such cases, fungal DNA can be extracted from FFPE tissue biopsy sections and amplified by PCR targeting *ITS* region of the fungal ribosomal RNA genes. Rooms et al. (38) diagnosed a case of disseminated emergomycosis caused by *Es. pasteurianus* in Uganda by using two broad-range fungal PCR assays targeting a region of the *28S* and the *ITS2* region, which was confirmed by phylogenetic analysis. While *ITS2* targets a diverse, non-coding region (200–300 bp) well represented in public databases, *28S* targets a more conserved coding region (330–350 bp), ideal for identification to genus level but not suitable for species resolution and is underrepresented in public databases (2, 39). Studies have shown that broad-range fungal PCR with sequencing is more successful in identifying fungal pathogens in pathology blocks, with even better performance by enhancing DNA recovery from tissue. However, amplification of fungal DNA from FFPE tissue is restricted by amplicon length, PCR inhibition, an excess of host DNA, and contaminating fungal DNA (39, 40).

### Serodiagnosis

There are no sensitive and specific serological tests or biomarkers for the diagnosis of emergomycosis. However, cross-reactivity has been observed with tests for other dimorphic fungal pathogens. Schwartz et al. (27) in a retrospective case series reported that two out of three patients of emergomycosis had positive urine *Histoplasma* galactomannan antigen and one out of four cases had positive 1,3-β-D-glucan test. A recent study by Maphanga et al. (41) also demonstrated cross-reactivity of *Histoplasma* galactomannan enzyme immunoassay in urine specimens of patients with *Es. africanus*. Further studies are required to evaluate the diagnostic and prognostic significance of various serological markers in emergomycosis.

## Antifungal Susceptibility Testing

The Clinical and Laboratory Standards Institute (CLSI) and the European Committee on Antimicrobial Susceptibility Testing (EUCAST) have established reference methods for susceptibility testing of yeasts (M27-A3) and molds (M38-A2) to the major classes of antifungal drugs which include the polyenes, azoles, and echinocandins. However, these reference methods have not been extended to include the dimorphic fungi leading to confusion as to whether susceptibility testing methods for yeasts or for molds should be followed (42). This issue is critical as the yeast and hyphal forms of dimorphic fungi exhibit different susceptibility patterns. Unfortunately, most antifungal studies continue to ignore the pathogenic yeast phase and instead, limit observation to mycelial-phase cells. Although conversion from mold to yeast phase increases the turnaround time, laboratory safety concerns are fewer with the yeast phase compared to the potentially hazardous mold phase. Hence, susceptibility testing with the yeast phase is recommended (37). There are no well-standardized methods for MIC determination of thermally dimorphic fungi. Antifungal susceptibility testing can be performed on yeast and mold phase isolates of *Emergomyces* spp. by a reference broth microdilution (BMD) method and the commercial Epsilometer test (E-test) method, as described by Maphanga et al. (37) The method follows the CLSI-approved standards for testing yeast and mycelial phases, but uses a larger-than-recommended inoculum for the latter (2.5 × 10^5^ CFU/ml, diluted 1:10 in Roswell Park Memorial Institute [RPMI] 1640 medium) and a prolonged incubation period of 7 days to facilitate growth and endpoint determinations (14, 39, 43).

## Treatment

Currently, there are four classes of antifungal drugs for the treatment of systemic mycoses: polyenes (amphotericin B), azoles (fluconazole, itraconazole, posaconazole, voriconazole, and isavuconazole), echinocandins (caspofungin, micafungin and anidulafungin), and antimetabolites (flucytosine). To date, there are no treatment guidelines for patients with emergomycosis. In absence of randomized controlled trials, it is recommended that treatment of disseminated emergomycosis should follow the Infectious Diseases Society of America guidelines for the management of endemic mycoses in immunocompromised persons (44).

In general, the dimorphic fungal pathogens exhibit a similar susceptibility profile for current antifungals with both amphotericin B and azoles (except fluconazole) showing potent activity (low minimum inhibitory concentrations; MIC) (42). The recommended regimen consists of amphotericin B (deoxycholate formulation 0.7–1.0 mg/kg daily or preferably, a liposomal formulation 3–5 mg/kg daily) for 1–2 weeks, followed by oral itraconazole (200 mg three times daily for 3 days and then 200 mg twice daily) for at least 12 months. Lifelong suppressive therapy with itraconazole (200 mg daily) may be required if immunosuppression cannot be reversed (44). The efficacy of different classes of antifungal drugs in the treatment emergomycosis are described below.

### Polyenes

Polyenes exert fungicidal activity by binding to ergosterol in the fungal cell membrane, resulting in its disintegration and the leakage of intracellular components, which subsequently leads to cell death (45). Amphotericin B has a broad spectrum of fungicidal activity and is recommended for the treatment of severe disseminated emergomycosis (3). Dukik et al. (42). evaluated the susceptibility patterns of 24 isolates from three clinically related genera, *Emergomyces, Adiaspiromyces*, and *Blastomyces* and reported that amphotericin B was the most active antifungal agent having the lowest MICs against all species, with geometric mean (GM) MIC and MIC ranges for *Emergomyces* being 0.049 and <0.016–0.25 μg/mL, respectively. Maphanga et al. (37) studied the MIC distribution of yeast and mold phases of 50 *Es. africanus* isolates and observed a low GM MIC of amphotericin B (Etest MICs 0.03 vs. 0.01 mg/L). Also, patients who received an antifungal regimen that included amphotericin B had a higher survival rate and better clinical outcome than those treated with a triazole alone. Similar findings were reported by Schwartz et al. (27) These observations highlight amphotericin B as the cornerstone in the management of disseminated emergomycosis.

### Azoles

Azoles act by inhibiting lanosterol 14α-demethylase, a key enzyme in ergosterol biosynthesis, resulting in depletion of ergosterol and accumulation of toxic 14α-methylated sterols in membranes (46, 47). Several studies have documented high MIC for fluconazole and therefore, less potent as compared to other azoles in the treatment of emergomycosis (17, 31, 37, 42). Schwartz et al. (27) observed that therapy with fluconazole was associated with increased mortality and poor outcomes. Interestingly, Moodley et al. (24) reported successful treatment of a case of disseminated emergomycosis caused by *Es. africanus* with 6 months of fluconazole monotherapy, as evidenced by complete resolution of skin lesions and clinical improvement. Among the newer triazoles, low MIC has been observed for itraconazole which makes it ideal for oral step-down phase following amphotericin B therapy (37, 42). However, due to non-linear pharmacokinetics, high intra-subject variability in plasma concentrations and risk of drug–drug interactions, therapeutic drug monitoring is mandatory in such patients (45–48). Dukik et al. (42) studied the *in vitro* susceptibility patterns of 11 isolates of *Emergomyces* spp. and reported that posaconazole had the lowest GM MIC values, followed by amphotericin B, itraconazole, voriconazole, and isavuconazole. A susceptibility study on yeast and mold phases of 50 *Es. africanus* isolates, also demonstrated low GM MIC value for posaconazole (37). Furthermore, a case of disseminated infection caused by *Es. pasteurianus* in Netherlands was successfully treated with 14 months of posaconazole therapy (18), indicating that posaconazole can be a possible treatment option for patients with disseminated disease. Isavuconazole is a novel triazole that has shown promise in the treatment of endemic mycoses in animal models (49, 50). However, studies have reported comparatively high MIC values against *Emergomyces* spp. (18, 42) and therefore, cannot be considered as a suitable treatment option in such cases.

Although itraconazole is commonly recommended for long-term treatment of endemic mycoses in HIV infected patients, caution should be exercised during concurrent administration with antiretroviral drugs, particularly protease inhibitors (PIs) and non-nucleoside reverse transcriptase inhibitors (NNRTIs) (51). Itraconazole being a substrate of CYP450 3A4 isoenzyme, PIs like lopinavir/ritonavir increase the serum levels of itraconazole resulting in toxicity (manifested by QT prolongation), while NNRTIs like nevirapine and efavirenz decrease its serum concentrations, leading to therapeutic failure. These potential drug-drug interactions limit their co-administration. However, integrase inhibitors like dolutegravir and raltegravir, which are currently recommended for the treatment of HIV infection are free from such adverse interactions and therefore, can be administered concurrently with itraconazole (52). In addition, the bioavailability of oral itraconazole is affected by gastric pH- bioavailability is enhanced when taken with food but reduced by 40% in the fasting state. AIDS-related hypochlorhydria also decreases its absorption significantly (53).

### Echinocandins

The echinocandins (caspofungin, anidulafungin, micafungin) inhibit the fungal 1,3-β-D-glucan synthase and depletes the fungal cell wall of 1,3-β-D-glucan, causing lysis of the fungal cell. However, they lack efficacy against the pathogenic phase of dimorphic fungal pathogens, including *Emergomyces* spp. (43) Maphanga et al. (37) studied the antifungal susceptibility pattern in 50 clinical isolates of *Es. africanus* and observed high GM MIC values for all three echinocandins tested. Similar results were obtained by Dukik et al. (42) for anidulafungin and micafungin.

## Prognosis

Disseminated emergomycosis is fatal if left untreated and patients who receive an antifungal regimen that includes amphotericin B are more likely to survive than those treated with a triazole alone. Monotherapy with either fluconazole or itraconazole are associated with poor outcomes. Schwartz et al. (27) in a retrospective case series of 54 patients with disseminated emergomycosis observed a case fatality rate of 48%, which included, among others, all of the patients that did not receive antifungal treatment.

## Future Prospects: What Is There in the Pipeline?

Considering the host toxicity profile of currently available antifungal drugs and the endogenous resistance of dimorphic fungi to the less toxic echinocandins, new and alternative antifungal drugs, preferably with novel modes of action (to avoid cross-resistance and/or cross-toxicities) need to be explored. Also, the availability of oral formulations would enable ambulatory treatment resulting in improved patient compliance and adherence to treatment. So far, none of the new antifungal agents have been evaluated for the treatment of emergomycosis. Some of the attractive antifungal candidates that might hold promise in the treatment of emergomycosis are summarized in Table 4.

**Table 4:** Antifungal drugs with novel targets in development and their spectrum of activity.

**Drug class**	**Antifungal agent**	**Mechanism of action**	**Target species**	**Trial phase and manufacturer**	**Advantages**
Polyene	Amphotericin B cochleate	Cochleate consists of a spiral structure made up of phosphatidylserine with phospholipid-calcium precipitates. It is stable against degradation by gastric acid, gets readily absorbed from GI tract and enters circulation where the calcium moiety is removed and the spiral formation opens up and releases the encapsulated drug into the target cell.	*Candida, Aspergillus, Cryptococcus*	Phase II !!break Matinas BioPharma	Oral formulation, less toxic, minimal drug-drug interactions
Azole	SUBA-itraconazole	Blocks CYP450 activity and inhibits ergosterol synthesis and cell membrane formation	*Histoplasma*, !!break *Blastomyces*, !!break *Aspergillus*	FDA approved !!break Mayne Pharma Ltd.	Increased bioavailability (173%) compared to conventional itraconazole
Orotomides	Olorofim	Inhibition of fungal dihydroorotate dehydrogenase and pyrimidine synthesis	*Aspergillus, Histoplasma, Blastomyces, Coccidioides, Talaromyces marneffei, Lomentospora*	Phase II !!break F2G Ltd.	Oral formulation, less toxic (fungal specific), active against multidrug resistant fungi
Phosphonooxymethylene	Fosmanogepix	Inhibition of fungal Gwt1 GPI anchor protein	*Candida* (except *C. krusei*), *Aspergillus, Fusarium, Scedosporium, Coccidioides*, some mucorales	Phase II !!break Amplyx Pharmaceuticals	Oral formulation, less toxic (fungal specific), broad spectrum antifungal activity
Polyoxins	Nikkomycin-Z	Inhibits chitin synthase and blocks fungal cell wall synthesis	*Histoplasma, Blastomyces, Coccidioides, Sporothrix*	Phase I !!break Valley Fever Solutions, Inc.	Synergistic action with echinocandins, orphan drug for coccidioidomycosis
Tetrazole	VT-1598	Blocks CYP51 activity and inhibits ergosterol synthesis and cell membrane formation	*Candida, Cryptococcus, Aspergillus, Rhizopus, Histoplasma, Blastomyces, Coccidioides*	Phase II !!break Mycovia Pharmaceuticals	Broad spectrum antifungal activity, longer half-life, minimal drug interactions (selective for fungal CYP51)
Celecoxib derivative	AR-12	Inhibition of fungal acetyl-CoA synthetase	*Candida, Cryptococcus, Blastomyces, Histoplasma, Coccidioides, Fusarium, Mucor, Lomentospora*	Phase I (oncology indications) !!break Arno Therapeutics	Repurposed drug with potent broad-spectrum antifungal activity

*GI, Gastrointestinal; CYP, Cytochrome P; GPI, Glycosylphospatidyl inositol; acetyl-CoA, acetyl coenzyme A*.

### Amphotericin B Cochleate

Amphotericin B cochleate (CAMB/MAT2203; Matinas BioPharma) is a new oral formulation belonging to the polyene class which is currently under Phase II clinical trial. Unlike other formulations, the cochleate form is stable against degradation in the gastrointestinal tract. Cochleate consists of a spiral structure made up of phosphatidylserine with phospholipid-calcium precipitates (54). Following oral administration, the cochleate is absorbed from the GI tract and enters circulation where the calcium moiety is removed and the spiral formation opens up and releases the encapsulated drug into the target cell. The limitations of cochleate formulations include high cost, stringent storage conditions (4°C), and precipitation during storage (55).

### SUBA-Itraconazole

The bioavailability of conventional oral itraconazole is limited by elevated gastric pH due to AIDS related hypochlorhydria, and use of proton pump inhibitors and H_2_ blockers. In order to improve the oral bioavailability, the drug has been optimized as super-bioavailability-itraconazole (SUBA-itraconazole; Mayne Pharmaceuticals), which is not affected by food or gastric pH (56). This new formulation utilizes the solid dispersion of the drug in a pH-dependent polymer matrix to enhance dissolution and absorption, thereby increasing oral bioavailability (173%) while reducing inter-patient variability (57). SUBA-itraconazole has been approved by the FDA for the treatment of blastomycosis, histoplasmosis, and aspergillosis (in patients intolerant or refractory to amphotericin B therapy). It is currently available as a 65 mg oral capsule, with a potential loading dose of 130 mg three times daily for the first 3 days, followed by a maintenance dose of 130 mg once daily.

### Olorofim (F901318)

Olorofim (F901318; F2G Ltd.), belonging to the class of orotomides, is a new antifungal drug under Phase III clinical trial that acts by inhibiting dihydroorotate dehydrogenase (58), a key enzyme in pyrimidine biosynthesis, which adversely affects fungal nucleic acid, cell wall and phospholipid synthesis, cell regulation and protein production (59). Olorofim has a good bioavailability and can be administered both orally and intravenously. It is widely distributed in tissues, such as kidney, liver, lung, and even brain (58). Besides its activity against *Scedosporium, Lomentospora*, and various cryptic species of *Aspergillus* (47, 60), it has been found to be effective against *Coccidioides* and other endemic fungi (61). The novel mechanism of action, broad spectrum of antifungal activity and absence of cross-resistance with other antifungal classes make Olorofim a promising candidate for the treatment of emergomycosis.

### Fosmanogepix (APX001)

Fosmanogepix (FMGX, APX001, Amplyx Pharmaceuticals) is a prodrug of manogepix that inhibits fungal enzyme Gwt1 and affects maturation and localization of GPI-anchored mannoproteins, leading to alterations of cell wall integrity, adhesion, and host immune evasion. The drug has excellent bioavailability following oral and intravenous administrations and is currently under Phase 2 clinical trials for the treatment of invasive fungal infections caused by *Candida, Aspergillus, Fusarium, Scedosporium*, and some mucorales (62). It has also demonstrated significant activity against *Coccidioides* spp. in murine model as evident by decreased fungal burden, halting systemic disease, and longer survival of FMGX-treated mice (63). Thus, considering its unique mechanism of action and broad spectrum antifungal activity, fosmanogepix is worthy of further evaluation for the treatment!!break of emergomycosis.

### Nikkomycin Z (VFS-1)

Nikkomycin Z (Valley Fever Solutions, Inc.) is a modified-nuceloside analog belonging to the class of polyoxins. It is a competitive inhibitor of chitin synthase that blocks fungal cell wall synthesis. It exhibits synergistic activity with the 1,3-β-D-glucan synthase inhibitors like echinocandins (47). Nikkomycin Z is currently in Phase I clinical trial and *in vitro* studies have demonstrated good efficacy against dimorphic fungal pathogens, such as *Histoplasma capsulatum* (64), *Blastomyces dermatitidis, Coccidioides immitis* (65), and *Sporothrix* spp. (66) Its action against *Emergomyces* spp. needs to be evaluated.

### Tetrazole (VT-1598)

Tetrazole antifungals are novel azole-like compounds that inhibit fungal lanosterol demethylase, resulting in decreased ergosterol synthesis and inhibiting cell membrane formation. Unlike currently available triazoles that interact with human CYP450 enzymes, tetrazoles exhibit high degree of selectivity for fungal CYP51 and consequently fewer drug-drug interactions (67). VT-1598 (Mycovia Pharmaceuticals) is currently in Phase II clinical trial and demonstrates activity against yeasts, molds, and endemic fungi (*B. dermatitidis, Coccidioides spp*., and *H. capsulatum*). Low MIC values and improved survival have been observed with VT-1598 against *Coccidioides* spp. in a murine model (68). Considering its broad-spectrum of activity against dimorphic fungi and favorable safety profile, VT-1598 might be a potential treatment option for emergomycosis.

### AR-12

AR-12 (Arno Therapeutics), a celecoxib derivative, was originally developed as an anticancer drug and is currently under phase I trial. Its antifungal activity is attributed to inhibition of fungal acetyl-CoA synthetase, causing depletion of acetyl coenzyme A (acetyl-CoA). It also downregulates host chaperone proteins and augments host immune response (46). The drug has demonstrated promising *in vitro* activity against *Candida, Cryptococcus, Mucor, Fusarium, Scedosporium*, and dimorphic fungi (*Blastomyces, Histoplasma*, and *Coccidioides*) (69). However, its clinical efficacy as an antifungal drug is yet to be ascertained.

## Conclusion

Emergomycosis is a fatal systemic fungal disease among immunocompromised patients in endemic regions and diagnosis is challenging, particularly in resource limited settings. A high index of suspicion is needed, especially in countries where tuberculosis and dimorphic fungal infections are endemic. In absence of consensus guidelines for the treatment of this condition, the IDSA recommendations for management of histoplasmosis and blastomycosis are being followed for emergomycosis. However, mortality still remains very high and the scenario is further complicated by drug-drug interactions, emergence of resistance and intolerance to the available antifungals. The newer drugs, such as amphotericin B cochleate, SUBA-itraconazole, olorofim, fosmanogepix, VT-1598 and AR-12 are a welcome addition to the antifungal armamentarium. Several of these agents have demonstrated good *in vitro* activity against endemic fungi like *Histoplasma, Blastomyces, Coccidioides*, and *Sporothrix* with better tolerability and safety profile. Future studies should focus on evaluating the efficacies of these novel antifungals against *Emergomyces* spp. which would help in optimizing management and improving patient outcomes.

## Author Contributions

ASa and ASh conceptualized the work, reviewed, edited, and validated the final version of the manuscript.

## Conflict of Interest

The authors declare that the research was conducted in the absence of any commercial or financial relationships that could be construed as a potential conflict of interest.
